# Intermittent Fasting Partially Alleviates Dietary Margarine-Induced Morphometrical, Hematological, and Biochemical Changes in Female Mice, but Not in Males

**DOI:** 10.1155/bri/2163104

**Published:** 2025-06-18

**Authors:** Viktoriia V. Hurza, Maria M. Bayliak, Myroslava V. Vatashchuk, Oksana M. Sorochynska, Maria P. Lylyk, Oleksandra B. Abrat, Dmytro V. Gospodaryov, Kenneth B. Storey, Volodymyr I. Lushchak

**Affiliations:** ^1^Department of Biochemistry and Biotechnology, Vasyl Stefanyk Precarpathian National University, Ivano-Frankivsk, Ukraine; ^2^Research and Development University, Ivano-Frankivsk, Ukraine

**Keywords:** every-other-day fasting, food consumption, inflammation, liver, metabolic health, sex differences, western diet

## Abstract

Margarine is a popular high-calorie component of the Western diet and was shown to be associated with the development of metabolic syndrome. Intermittent fasting (IF) is an effective approach to improve health and prevent metabolic disorders. This study aimed to investigate the effects of margarine consumption, both *ad libitum* and in combination with IF regimens, using young C57BL/6J mice of both sexes. Female mice fed margarine *ad libitum* as a supplement to the standard diet showed significant body mass gain, reduced food intake, lower blood paraoxonase activity, and higher lipid peroxide (LOOH) levels, along with higher activities of antioxidant enzymes in the liver. Margarine-fed males showed higher food intake and had lower blood triacylglycerol levels, higher LOOH levels in adipose tissue, and lower LOOH levels in the liver than their control counterparts. When a margarine-supplemented diet was provided to mice with an IF regimen, males gained body mass faster and experienced severe metabolic changes, including elevated fasting blood glucose levels, higher total leukocyte count, triacylglycerol accumulation, and reduced glycogen levels in the liver compared to their margarine *ad libitum* counterparts. Females treated with margarine + IF showed a partial improvement in metabolic status and a decrease in proinflammatory markers compared to the group receiving margarine *ad libitum*. Hence, responses to the diets were sex-specific. Females that consumed margarine *ad libitum* had higher metabolic sensitivity than males. Meanwhile, IF provided some protective effects in females but worsened metabolic outcomes in males when combined with a high-fat margarine diet.

## 1. Introduction

Metabolic syndrome (MetS) is one of the most common diseases and is often associated with obesity [1]. Changes in metabolism are among the main parameters that characterize MetS. Clinically, this is manifested in elevated blood glucose and triacylglycerol (TAG) levels, insulin resistance, increased markers of inflammation, and oxidative stress [2, 3]. The syndrome significantly increases the risk of cardiovascular diseases and type 2 diabetes. Consumption of foods rich in fats and carbohydrates is among the possible causes of MetS development [2, 3].

The role of trans-fatty acids (TFAs) in the development of MetS is controversial. It has been found that TFAs of different origins may have opposite effects on human health [4]. Generally, there are two types of TFAs: those formed due to metabolism in living organisms and those formed due to industrial processing [5]. Data on the association between consumption of TFAs and health risks are controversial. Some studies suggest that consumption of TFAs from animal sources reduces the risk of developing MetS and cardiovascular diseases [6, 7], whereas others showed that consumption of industrially produced TFAs is more dangerous for health than consumption of natural TFAs [5, 8–10]. However, no differences between the adverse effects of natural and industrially produced TFAs or their anti-inflammatory properties have been reported [4, 11].

Industrially produced TFAs are formed during the partial hydrogenation of vegetable oils, a process of converting liquid vegetable oils into solid forms [5]. Margarine is one of the food products that includes hydrogenated vegetable fats and is often used in baking as an integral part of the Western diet [5, 12]. While dietary intake of industrial TFAs has been linked to inflammation, type 2 diabetes, and cardiovascular disease, understanding the impact of dietary fats such as margarine or butter on human health remains challenging due to their complex composition and the individual effects of their components on metabolism [13]. Saturated and unsaturated lipids are the main components of margarine, with a small content of TFAs and several other secondary ingredients (emulsifiers, salt, flavors, preservatives, acidity regulators, etc.). Some studies report that a margarine-containing diet might be associated with MetS development since it may cause changes in serum metabolome in humans [13], promote expression of genes whose products are involved in lipogenesis [14], induce inflammation in white adipose tissue [15], and/or increase body mass (BM) in rats [16]. Moreover, margarine was shown to induce changes in endocannabinoid tone in female rats that can contribute to the development of binge eating [17]. At the same time, there is a lack of research that comprehensively assesses the health effects of margarine, in particular, the dependence of its effects on sex and feeding regimen.

Intermittent fasting (IF), in particular every-other-day fasting, can be an effective approach to managing MetS symptoms [18–21]. It has been shown that obese mice on a high-fat diet with an IF regimen lost BM and had improved glucose tolerance and dyslipidemia [22, 23]. Park et al. [20] reported that IF reduced BM, body mass index (BMI), body mass fat, and total cholesterol in overweight people. In patients with nonalcoholic fatty liver disease, a positive effect of IF was manifested in improving dyslipidemia with a significant decrease in serum TAG [18]. The IF regimen has been shown to have mild modulating effects on bioenergetics and oxidative stress parameters in young mice fed a standard rodent chow [24–26].

Given that margarine consumption may contribute to metabolic dysregulation and inflammation, and IF has demonstrated potential benefits in mitigating these effects, we sought to investigate whether IF could counteract the adverse metabolic impacts of a margarine-supplemented diet. This combination is particularly relevant because margarine, as a high-fat food, may exacerbate oxidative stress and inflammation, key contributors to MetS, while IF has been shown to reduce these markers. In particular, IF can lower proinflammatory cytokines such as interleukin-1β (IL-1β) [27], which is elevated in MetS and disrupts insulin secretion, contributing to pancreatic β-cell dysfunction [28, 29]. Additionally, IF may modulate oxidative stress by reducing reactive oxygen species (ROS) production, which is increased in obesity and other MetS-related conditions [30–32]. Excessive ROS can damage cellular components, including lipids in membranes, leading to further metabolic dysfunction [31–33].

Our previous study showed that the combination of IF with a standard diet caused metabolic stress in healthy young mice [34]. Munhoz et al. [28] found that administration of IF to young female rats reduced their BM and food intake but increased body fat and plasma insulin levels while decreasing muscle mass. These findings suggest that the effects of IF may depend on the dietary context and the metabolic state of the individual. Given the ambiguous effects of IF on young animals and the lack of studies comparing its impact across sexes, we aimed to evaluate how margarine consumption, alone or combined with IF, affects metabolic, inflammatory, and oxidative stress markers in both male and female mice. This approach will clarify whether IF can act as a protective strategy against margarine-induced metabolic disturbances and whether these effects are sex-specific.

The present study aimed to evaluate whether margarine as a supplement to a standard diet affected the development of MetS-related metabolic changes in male and female mice. Given that margarine may lead to binge eating [17], we assessed these parameters in mice that had a choice to eat either basic chow or margarine under two feeding regimens: *ad libitum*, where mice had constant free access to chow and margarine, and IF, when free access to chow with margarine was provided for 24 h followed by 24 h of fasting (i.e., every-other-day IF). By examining key metabolic markers such as proinflammatory cytokines (IL-1β), antioxidant enzyme activities, and levels of oxidative lipid damage, we aimed to provide a comprehensive understanding of the sex-specific responses to these dietary interventions.

## 2. Materials and Methods

### 2.1. Animal Husbandry and Experimental Design

**Figure 1:**
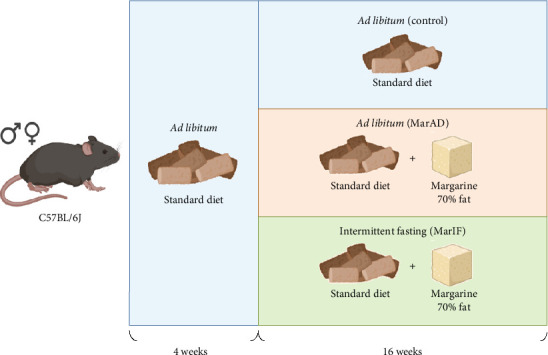
Experimental design. One-month-old mice were randomly divided into three groups: (i) the control group was fed *ad libitum* a standard food (control), (ii) margarine group was fed *ad libitum* standard food supplemented with margarine (MarAD), and (iii) the margarine + intermittent fasting (MarIF) group was also fed standard food supplemented with margarine, but with an alternating schedule of 24 h of feeding followed by 24 h of complete fasting.

C57BL/6J mice were used for the experiment. Mice were bred in the Department of Biochemistry and Biotechnology vivarium at Vasyl Stefanyk Precarpathian National University. One-month-old mice were randomly divided into three groups and subjected to experimental diets (Figure 1).

Mice in the first group (control) continued to consume the basic rodent chow (“Rezon-1” PE, Kyiv, Ukraine) *ad libitum* for 16 weeks. The chow contained 21.8% protein, 4.8% fat, 69.1% carbohydrates, and 3.9% fiber. Each group had unlimited access to water. The animals were kept on experimental diets for the next 16 weeks. The second and third groups were supplemented with margarine containing 70% fat (saturated fats—12.9–18.2 g/100 g, unsaturated fats—57.1–51.8 g/100 g) (Soniachnyi Shchedryk, Olkom TM, Ukraine) in addition to the standard chow, also for the next 16 weeks. The margarine was given in a separate bowl, so mice had a choice to eat the standard food alone or the margarine. The second group (margarine *ad libitum* group abbreviated as MarAD) had unlimited access to a standard diet and margarine, whereas the third mouse group (margarine + IF—also called IF—abbreviated as MarIF) was subjected to every-other-day IF. The IF regimen consisted of *ad libitum* food availability for 24 h, followed by complete fasting for the next 24 h. All groups had unlimited access to water. Each mouse group was housed in two cages with three mice of the same sex in each cage. In total, six males and six females were used in each experimental group. The detailed composition of the standard rodent chow and margarine is given in the Supporting information, Supporting Table 1, and Supporting File 1, respectively. Standard food and water were replaced weekly at 9 a.m. Portions of margarine were replaced every other day. For margarine-eating mouse groups, food was always provided or withdrawn at 9 a.m. The mice were on these diets for 16 weeks under a 12-h light/dark cycle (6 a.m./6 p.m.) at a temperature of 22 ± 2°C and a humidity of 50%–60%.

All procedures were approved by the Animal Experimental Committee of Vasyl Stefanyk Precarpathian National University following Directive 2010/63/EU of the European Parliament and the Council of the European Union for the protection of animals used for scientific purposes.

### 2.2. Physiological Parameters and Tissue Sampling

BM of the mice, along with the amounts of water and standard food consumed, was recorded once a week. The amount of margarine eaten was determined every 2 days when it was replaced with a new portion. The measurements were taken between 9 and 10 a.m. Before sampling, mice were fasted for 16 h and then anesthetized with carbon dioxide. In anesthetized mice, blood was collected from the retro-orbital sinus [34], and then the body length (in centimeters) of the mice was measured from the tip of the nose to the base of the tail on the abdominal side. This measurement, along with BM, was used to calculate the BMI (g/cm^2^) and the Lee obesity index [29]. Mice were euthanized by cervical dislocation, and visceral adipose tissue and liver samples were taken from the mice and frozen for further assessment, as described in our previous study [24].

### 2.3. Blood Leukocyte Formula and Determination of Biochemical Parameters in Blood Plasma

After collection, blood samples were divided into two parts. About 10 μL of blood was used for blood smear preparation. Blood smears were stained using the Pappenheim method for microscopic analysis of leukocytes. First, the blood smears were placed in May–Grünwald stain for 40 min. After washing with water, the smears were placed in Giemsa stain for 20 min. The prepared slides were examined under a light microscope using an immersion objective at a magnification of 1000x [30, 34].

Another portion of the blood was centrifuged at 1500 g, 15 min, 4°C to obtain plasma. Plasma was collected in new tubes and stored at −85°C until used to determine activities of paraoxonase (PON) and myeloperoxidase (MPO), as well as levels of total cholesterol, TAGs, and glucose.

The activity of PON was determined using 4-nitrophenylacetate as a substrate by monitoring the formation of 4-nitrophenol at 412 nm with a spectrophotometer Spekol 211 (Carl Zeiss, Jena, Germany) [33]. The activity of MPO was determined by the oxidation of 3,3′,5,5′-tetramethylbenzidine dihydrochloride with hydrogen peroxide, and optical density read at 450 nm in a Multiskan MCC/340 microplate reader (Labsystems Diagnostics Oy, Vantaa, Finland) [32, 35]. Enzyme activity was expressed as mmol/L. The levels of TAGs, free glucose, and total cholesterol in the blood plasma were determined using respective diagnostic kits from Felicit-Diagnostics Ltd. (Dnipro, Ukraine) according to the kit instructions.

### 2.4. Determination of IL-1β Levels

Samples of frozen adipose tissue were homogenized in cold lysis buffer (50 mM Tris pH 7.5, 150 mM NaCl, 0.1% Triton X-100) with 20 mg/mL EDTA-free protease inhibitor cocktail SIGMA FAST (Merck KGaA, #S8830) and incubated for 2 h at 4°C for cell lysis. After centrifugation at 16,000 × g for 15 min at 4°C, the supernatants were collected and stored at −80°C until further analysis. In the supernatants, soluble protein levels were determined, and then samples were diluted 1:1 v:v with phosphate-buffered saline (PBS), and 250 ng of protein per well was added to a microtiter plate. Blood plasma was diluted fivefold in PBS, and 100 μL of diluted plasma was added per microplate well for each sample. Plates containing samples from adipose tissue and blood plasma were incubated at 4°C overnight and for 2 h at room temperature, respectively, to attach protein. After incubation, the plates were washed three times with 100 μL of PBS. For blocking nonbound places, 200 μL of 4% bovine serum albumin (BSA) in PBS was added to each well and incubated for 2 h at room temperature. Subsequently, the plates were washed three times with PBS. A solution of primary anti-interleukin-1β antibody (Abcam, #ab9722) was added to the wells and incubated for 2 h at room temperature. The primary antibody powder was initially diluted with distilled water to a concentration of 100 μg/mL and then further diluted in 4% BSA (1:100). After incubation and washing, secondary anti-rabbit immunoglobulin G was conjugated with horseradish peroxidase (HRP) (Cell Signaling Technology, Inc. #7074S), diluted in 4% BSA (1:2500), and added to each well and incubated for 2 h at room temperature. After washing, 100 μL of 3,3′,5,5′-tetramethylbenzidine dihydrochloride, a substrate for HRP, was added to the samples. The samples were then incubated at 37°C for 22 min until a blue color appeared. After incubation, the reaction was stopped by the addition of 100 μL of 2 M H_2_SO_4_, and optical density was determined at 450 nm in a Multiskan MCC/340 microplate reader according to the average absorption value of the control group.

### 2.5. Determination of Lipid Peroxide Levels

For the determination of concentrations of lipid peroxides (LOOH), frozen liver and adipose tissue samples were weighed and homogenized in 96% ethanol (1:10) using a Potter-Elvehjem homogenizer. Homogenates were centrifuged to obtain supernatants at 9240 × g, 10 min, 4°C in an Eppendorf 5415 R centrifuge (Hamburg, Germany). The assay is based on the oxidation of Fe(II) ions by lipid peroxides at acidic pH in the presence of xylenol orange, which binds Fe(III). Upon binding of oxidized iron with xylenol, an orange complex is formed with a maximum absorption at 580 nm. Cumene hydroperoxide was used as an internal standard [36, 37]. The results were expressed in nanomoles of cumene hydroperoxide equivalents per gram of wet tissue mass.

### 2.6. Determination of Activities of Antioxidant and Glycolytic Enzymes

For the determination of activities of glycolytic enzymes, frozen liver samples were homogenized in cold 50 mM imidazole lysis buffer (pH 7.5) containing 0.5 mM EDTA, 20 mM NaF, 150 mM KCl, 1 mM dithiothreitol, and 1 mM of protease inhibitor phenylmethylsulfonyl fluoride (PMSF). Activities of phosphofructokinase (PFK) and pyruvate kinase (PK) were measured in coupled reactions as described previously [25]. For the determination of antioxidant and related enzymes, samples of frozen liver tissue were homogenized in 50 mM potassium phosphate buffer (рН 7.0) with 0.5 mM EDTA and 1 mM PMSF. Activities of superoxide dismutase (SOD), catalase, glutathione peroxidase (GPx), glutathione-S-transferase (GST), and glucose-6-phosphate dehydrogenase (G6PDH) were measured by spectrophotometric methods described in our previous studies [25, 38].

### 2.7. Assay of Glycogen and TAG Levels in the Liver

Levels of glycogen and TAG in the liver were measured as described earlier [32]. Briefly, glycogen levels were assayed by glycogen conversion to glucose via the action of amyloglucosidase (Sigma-Aldrich, #10115; 0.56 U/μL). Levels of glucose produced were measured using a colorimetric glucose oxidase kit from Felicit-Diagnostics Ltd. (Dnipro, Ukraine), following the manufacturer's instructions. For determination of TAG levels, frozen liver samples were homogenized in PBS buffer containing 0.05% Triton X-100, incubated at 37°C for 15 min, and centrifuged. TAG levels in the resulting supernatants were then measured using a diagnostic kit from Felicit-Diagnostics Ltd. (Dnipro, Ukraine) according to the manufacturer's instructions.

### 2.8. Statistical Analyses

Statistical analysis was performed using R software (Version 4.3.1 for Windows). Data for the average daily food consumption and average daily caloric intake were evaluated by one-way analysis of variance (ANOVA). Other data were analyzed by two-way ANOVA to compare the parameters at two factors (diet and sex), followed by multiple comparisons using Duncan's new multiple range test implemented in R (package *DescTools*). Visualization was performed in GraphPad Prism Version 8.3.1 for Windows (GraphPad Software, Boston, Massachusetts, USA, https://www.graphpad.com). Detailed statistical analysis is given in the Supporting Table 2.

## 3. Results

### 3.1. Body Mass and Food Consumption

**Figure 2:**
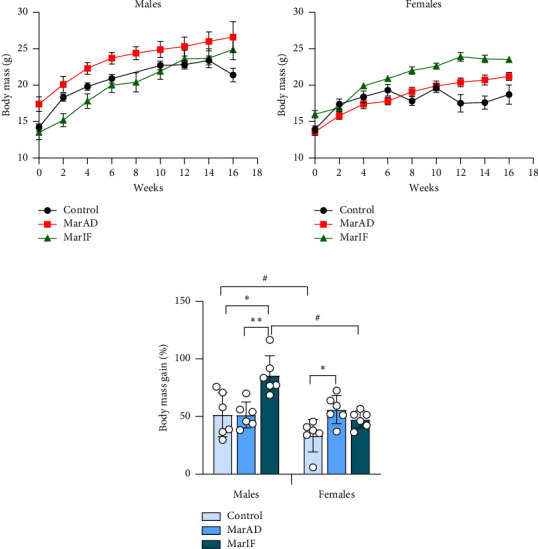
Changes in body mass in mice fed a standard food *ad libitum* (control), margarine-supplemented food *ad libitum* (MarAD), and margarine-supplemented food with an IF regimen for 16 weeks (MarIF). In each group, mice were housed in two cages, with three mice in one cage (*n* = 6 mice per group). Males and females were kept in separate cages. Dynamics of absolute body mass in males (a) and females (b), and percentage body mass gain at the end of the experiment (c). ^∗^Significantly different from the value in the corresponding control group with *p* ≤ 0.05. ^∗∗^Significantly different from the values in the corresponding MarAD group with *p* ≤ 0.05. ^#^Significantly different between sexes with *p* ≤ 0.05.

BM of males and females increased in all groups throughout the experiment throughout the entire period (Figures 2(a), 2(b)).

Since the BM of the mice in different groups varied slightly at the start of the experiment due to random assignment, we calculated the BM gain during the experiment as a percentage of the initial BM.  Figure 2(c) shows the percentage of BM gain in experimental mice. Males on the MarAD diet showed no change in BM gain, but those on MarIF gained more BM than controls. In females, only the group fed MarAD showed a significantly higher BM compared to the control group. Notably, although control females had 35% lower BM than control males, no differences in BM were observed between the sexes in the margarine-fed groups. Despite some differences between the groups, BMI and the Lee obesity index remained within the physiological range for rodents (Supporting Figs.  1-2).

**Figure 3:**
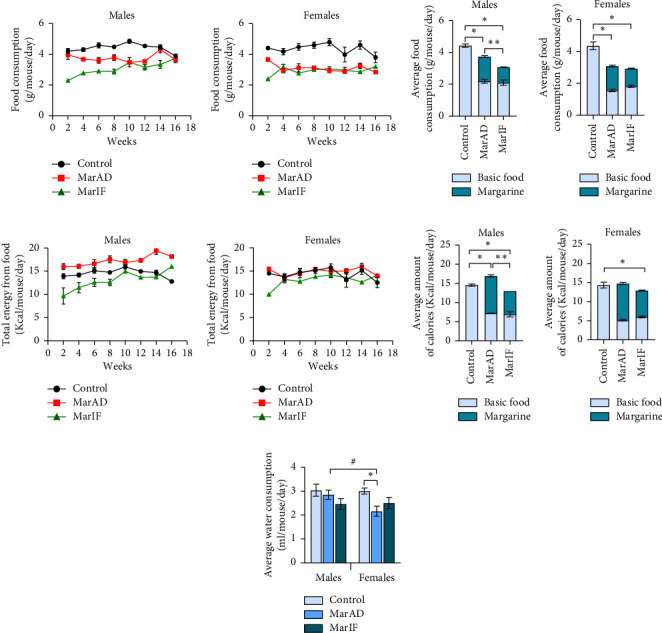
Food and energy consumption by mice fed a standard food *ad libitum* (control), margarine-supplemented food *ad libitum* (MarAD), and margarine-supplemented food with an IF regimen (MarIF). Dynamics of daily food consumption (a, b), average daily food consumption (basic food is marked in blue, margarine is marked in dark blue) (c, d), dynamics of daily energy intake (e, f), average daily number of calories consumed (calories from basic food is marked in blue, and calories form margarine is marked in dark blue) (g, h), and average daily water consumption (i). Statistical information is shown in Figure 2.

On the diets with margarine, individuals of both sexes consumed less chow than their control counterparts (Figures 3(a), 3(b)). Males in the MarAD and MarIF groups consumed 15% and 30% less total food, respectively, than their control counterparts. Females in both margarine groups consumed approximately 30% less total food than the control group (Figures 3(c), 3(d)). Thus, the MarIF regimen decreased total food intake in males but not in females as compared to the *ad libitum* margarine diet. Comparison of food consumption between the sexes showed no difference in the control groups, but females in the margarine-fed groups consumed less food than their male counterparts. It should be noted that females preferred margarine-containing food over basic food more than males when food was provided *ad libitum* (i.e., mice had permanent access to food). Specifically, margarine accounted for 42% and 60% of the total food consumed by males and females, respectively. Indeed, in the MarAD groups, males and females consumed more than twice as much basic food compared to their control counterparts. Both sexes of the MarIF group ate less margarine than their MarAD counterparts. At the same time, mice in both the MarAD and MarIF groups consumed similar amounts of the basic chow food. Males that consumed MarAD received 19% more calories from food than those on the control diet (Figures 3(e), 3(f)), whereas no such difference was observed in females (Figures 3(g), 3(h)). At the same time, males and females of the groups fed MarIF had lower total energy intake, by 23% and 12%, respectively, compared to the respective MarAD groups, and by 10% lower food intake compared to the control mice.

The percentage of calories derived from fat was within 72% for MarAD groups and 62% for MarIF groups in both sexes, compared to 16% in the control mice on the basal diet (Supporting Fig.  4). Females on the margarine-supplemented diet consumed 28% less water than the control mice, whereas margarine-fed males tended to have lower water intake compared to the control (Figure 3(i)).

### 3.2. Blood Parameters

**Figure 4:**
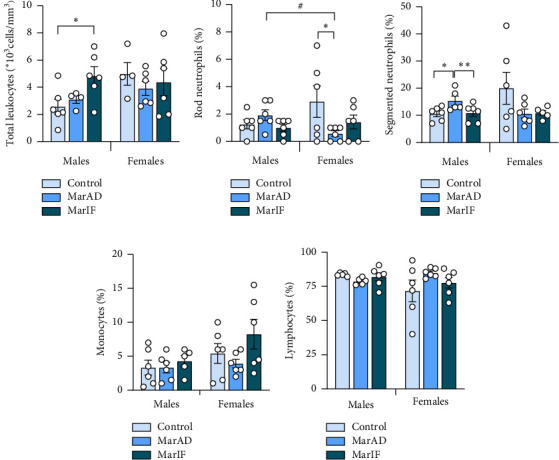
Total and differential leukocyte counts in mice fed a standard food *ad libitum* (control), margarine-supplemented food *ad libitum* (MarAD), and margarine-supplemented food with an IF regimen (MarIF). The number of total leukocytes (a) and relative counts of band neutrophils (b), segmented neutrophils (c), monocytes (d), and lymphocytes (e). Statistical information is shown in Figure 2.

Total leukocyte count and differential leukocyte count (leukocyte formula) are important indicators of clinical blood diagnostics. In males, the total number of blood leukocytes did not differ between the MarAD and control groups but was 87% higher in the MarIF group compared to the control group (Figure 4(a)). In females from both margarine-supplemented groups, the total leukocyte counts were similar to the values of the control group. Some cell types in the leukocyte formula in both males and females fed MarAD and MarIF differed from ones in the control group (Figures 4(b), 4(c), 4(d), 4(e)). The relative percentage of segmented neutrophils was 31% higher in MarAD males compared with the control group. The percentage of band neutrophils was 80% lower in MarAD females compared to controls. Other types of leukocytes showed no significant changes between the control and MarAD groups of both sexes. Margarine-fed females had 70% fewer band neutrophils than the respective males (Figure 4(b)). In the MarIF groups of both sexes, no differences in leukocyte formula were observed compared to the control groups.

**Figure 5:**
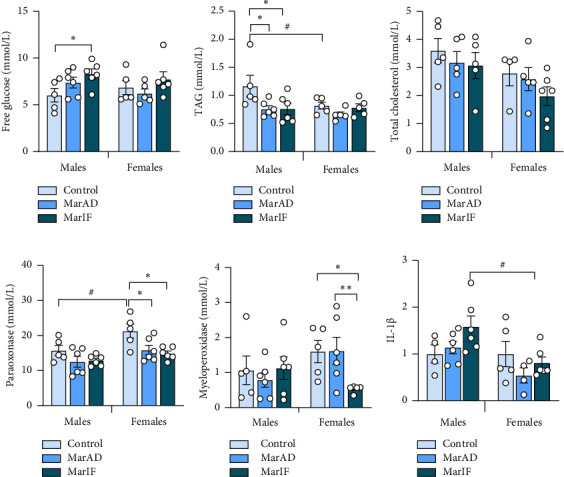
Blood biochemical parameters in mice fed a standard food *ad libitum* (control), margarine-supplemented food *ad libitum* (MarAD), and margarine-supplemented food with an IF regimen (MarIF). Levels of free glucose (a), triacylglycerols, TAG (b), total cholesterol (c), paraoxonase activity (d), myeloperoxidase activity (e), and interleukin-1β, IL-1β level (f). Statistical information is shown in Figure 2.

Fasting blood glucose levels were virtually the same in all studied groups, with the exception of the MarIF male group, which showed a significantly higher blood glucose level (by 37%) as compared to the control mice (Figure 5(a)).

MarAD and MarIF males had 35% lower plasma TAG levels compared to the control males (Figure 5(b)). In females, TAG levels did not differ between experimental groups. Plasma total cholesterol levels were virtually the same in all mouse groups (Figure 5(c)).

Blood PON activity in males did not differ among the three groups. However, the female MarIF group possessed 30% lower PON activity than control females. In control groups, PON activity was 34% higher in females than in males (Figure 5(d)). The activity of MPO was virtually the same in all three groups of males. MarIF females showed about 67% lower activity than the control and MarAD groups (Figure 5(e)).

In both sexes, IL-1β levels in blood plasma did not differ among the three groups. Comparing sex effects, only MarIF females had 49% lower levels than their male counterparts (Figure 5(f)).

### 3.3. Adipose Tissue IL-1β and Lipid Peroxides

**Figure 6:**
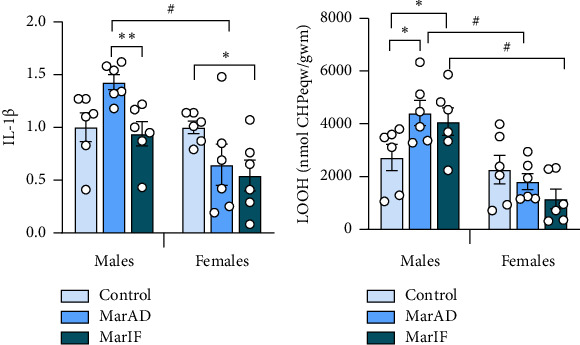
IL-1β levels and lipid peroxide (LOOH) levels in adipose tissue of mice fed a standard food *ad libitum* (control), margarine-supplemented food *ad libitum* (MarAD), and margarine-supplemented food with an IF regimen (MarIF). Levels of interleukin-1β, IL-1β (a), and lipid peroxides, LOOH (b). Statistical information is shown in Figure 2.

Figure 6(a) shows the levels of proinflammatory IL-1β in adipose tissue. In males, the MarIF group showed a 34% lower level of IL-1β compared to the MarAD group. In females, the MarIF group had 45% lower IL-1β levels than the control group. Finally, the female MarAD group showed a 55% lower level than the control group.

In the adipose tissue of males, the levels of lipid peroxides (LOOH) were 62% and 50%, higher in the MarAD and MarIF groups than in controls, respectively, whereas females showed no difference between groups for LOOH levels (Figure 6(b)). Interestingly, LOOH levels in MarAD and MarIF females were 59% and 72% lower, respectively, compared to males on the same diets.

### 3.4. Biochemical Parameters of the Liver

**Figure 7:**
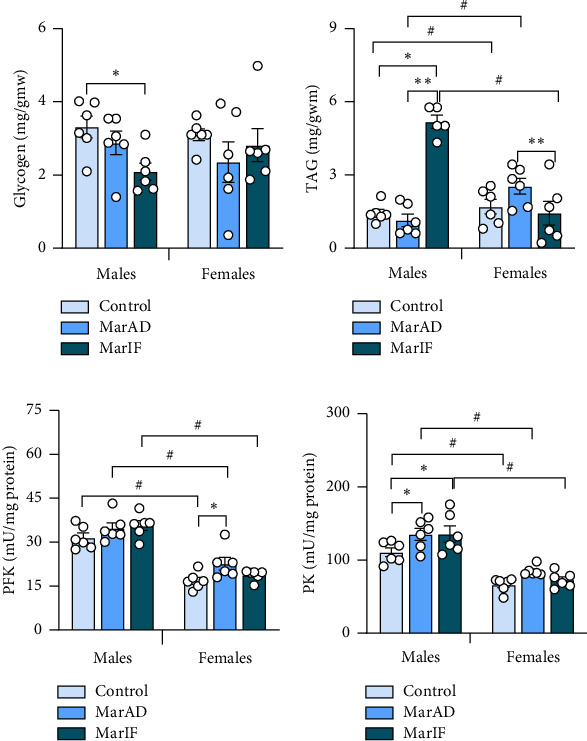
Energy metabolism indicators in the liver of mice fed a standard diet (control), margarine-supplemented food ad libitum (MarAD), and margarine-supplemented diet with an IF regimen (MarIF). Levels of glycogen (a), triacylglycerols (TAG) (b), and activities of phosphofructokinase (PFK) (c), and pyruvate kinase (PK) (d). Statistical information is shown in Figure 2.

In the liver of the MarIF male group, glycogen levels were 37% lower than in the control group, whereas no differences in this parameter were observed among the female groups(Figure 7(a)). The male MarIF group had about 3.5-fold higher TAG levels than the controls and the MarAD group. The MarIF females had a 44% lower TAG level than the MarAD group (Figure 7(b)). The activities of PFK in males did not differ among the three groups, whereas in females, the MarAD group showed 59% higher PFK activity than the other groups. Finally, all three groups of females possessed significantly lower PFK activity than respective males (Figure 7(c)). In MarAD and MarIF males, the activities of PK were 22% and 23% higher than in the control group, respectively, whereas no difference in PK activity was found between female groups. All three experimental groups of males showed higher PK activities than the respective females (Figure 7(d)).

**Figure 8:**
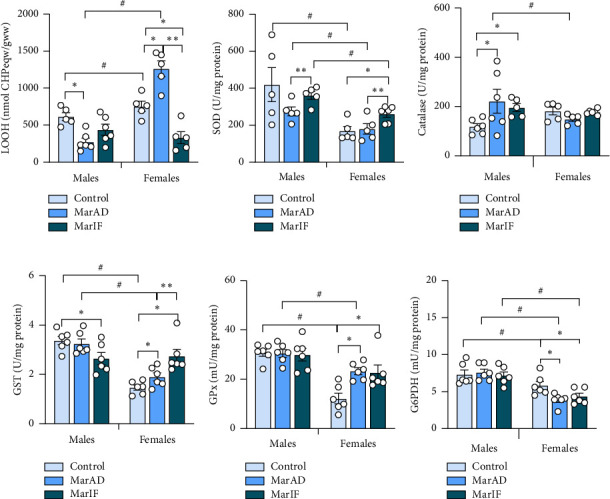
Markers of oxidative stress and antioxidant defense in the liver of mice fed a standard food *ad libitum* (control), margarine-supplemented food *ad libitum* (MarAD), and margarine-supplemented food with an IF regimen (MarIF). Lipid peroxide (LOOH) levels (a), activities of superoxide dismutase, SOD (b), catalase (c), glutathione-S-transferase, GST (d), glutathione peroxidase, GPx (e), and glucose-6-phosphate dehydrogenase, G6PDH (f). Statistical information is shown in Figure 2.

Next, we evaluated processes related to the homeostasis of ROS in the liver. In males, the MarAD group showed a 55% lower hepatic level of LOOH compared to the controls. In contrast, females in the MarAD group had 67% higher LOOH levels. In comparison, the MarIF females showed 56% and 74% lower LOOH levels than the controls and MarAD females, respectively (Figure 8(a)). In addition, control and MarAD females had higher LOOH levels than their respective male groups (Figure 8(a)).

MarAD regimen did not affect the activity of SOD in both sexes. SOD activity was 32% higher in MarIF males and 45% higher in MarIF females compared to their respective MarAD groups. In females, the MarIF group showed SOD activity that was 54% higher than in the control group, and all three groups of females showed lower SOD activities than their male counterparts (Figure 8(b)). The activity of catalase in the MarAD male liver was 87% higher than in the controls. MarIF males possessed 65% lower catalase activity than the MarAD group. In females, catalase activity did not differ between groups. However, catalase activity in the MarAD females was 33% lower than in the MarAD male group (Figure 8(c)). Hepatic GST activity in males was 23% lower in the MarIF group versus controls. In contrast, females showed increased GST activity, 30% higher in MarAD, and 88% higher in MarIF groups compared to controls, with MarIF females also exhibiting 44% higher activity than MarAD females. Finally, control and MarAD males showed higher GST activity than their female counterparts (Figure 8(d)). No differences in hepatic GPx activities were found between male groups. MarAD and MarIF females showed 91% and 86% higher GPx activities, respectively. In addition, control and MarAD males demonstrated higher GPx activities than their female counterparts (Figure 8(e)). No differences in G6PDH activity were observed among male groups. However, female MarAD and MarIF groups exhibited 34% and 26% lower G6PDH activity than controls, respectively. Importantly, males consistently showed higher G6PDH activity than females across all groups (Figure 8(f)).

## 4. Discussion

### 4.1. Female Mice Are More Sensitive to MarAD Diet

Mice in the control and all experimental groups gained BM during the experiment. This was expected because we used very young (one-month-old) mice at the beginning of the experiment. This is consistent with other studies [10, 34, 39]. Most studies related to obesity have used males who develop obesity signs well [10, 19, 39, 40]. By contrast, female mice have been shown to resist metabolic changes induced by a high-fat diet more than males [41, 42]. However, some studies have shown that consumption of a high-calorie diet containing margarine increased BM in male mice [43] and rats, in both males [44] and females [17]. In our study, the MarAD diet did notaffect BM gain in male mice but induced a significant BM gain in female mice compared to the standard diet (Figure 2(c)). The discrepancy with the data from the above studies may be due to the feeding regimen. In the study by Zhu et al. [44], margarine was mixed with a standard chow and fed to male rats. In the study of Satta et al. [17], female rats were supplemented with margarine in separate dishes, having a choice between foods. In our case, mice of both sexes could choose between eating the standard diet or margarine, and females preferred eating margarine. Choosing between low-calorie standard and high-calorie margarine foods can affect the amount of food consumed and the number of calories consumed. Mice in control groups consumed food amounts typical for this strain [45]. Both males and females consumed less total food when margarine was added to the basic diet. Despite reduced food consumption, males in the MarAD group consumed more calories than their control counterparts. That supports the idea that margarine can induce binge eating. Herewith, since margarine-fed females demonstrated a greater decrease in food intake than males, they did not differ from control females in energy intake. At the same time, MarAD females consumed a similar amount of calories from fat (margarine) as respective males (Supporting Figs.  4C, D). Hence, the BM increase in MarAD females cannot be explained by higher margarine intake or higher calorie intake. Some studies found that saturated fats led to more abdominal fat accumulation in female mice than in males due to a loss of estrogen levels [46, 47]. Therefore, we can suppose that intensive BM gain in females (Figure 2(c)) who consumed margarine continuously could be connected with decreased estrogen levels. However, there are not enough data to state about obesity development in margarine-fed mice because indices of obesity (BMI, Lee obesity index) were within the physiological range and only tended to be higher in females fed a margarine diet. At the same time, consumption of a high-calorie diet is not always accompanied by the development of visible obesity. In particular, if there is a lack of protein or certain amino acids in a high-calorie diet, mice may not develop obesity but display metabolic perturbations [32, 48]. In our study, mice consumed less basic food on margarine-supplemented diets and thus received lower amounts of protein. This can explain the absence of a significant increase in BM in these mice.

Blood biochemical and immunological parameters, in general, were not significantly affected by the MarAD diet, except TAG levels and PON activity. Most previous studies reported an increase in TAG levels on high-calorie diets [32, 49] or no differences [8] or a decrease in TAG levels in diet-induced obese mice due to dyslipidemia development [50, 51]. PON is an enzyme that is associated with high-density lipoproteins (HDL) and can neutralize lipid peroxides, thereby preventing the oxidation of lipoproteins [33, 52]. According to previous studies, PON activity decreases when acute inflammatory processes occur. Its activity inversely correlates with the activity of MPO, a proinflammatory defensive enzyme released from neutrophils when the latter are destroyed [33, 49, 51]. We did not observe changes in blood MPO in margarine-fed mice (Figure 5(e)) despite minor changes in the relative count of neutrophils in the leukocyte formula. No significant changes in total leukocyte count and plasma IL-1β levels were found in margarine-fed mice. This can suggest no development of systemic inflammation. However, neutrophils can migrate to the sites of inflammation and exert an immune response there, which leads to the release of ROS at the site of inflammation [53, 54]. Therefore, there is a possibility that, in our case, neutrophils might be translocated to the tissues in which inflammation began, in particular, visceral adipose tissue, as discussed above. Decreased PON activity in margarine-fed males may be due to impaired HDL synthesis or inactivation by free radicals or S-glutathionylation during oxidative stress [52].

The current study revealed that MarAD led to higher levels of lipid peroxides in the visceral adipose tissue of males but not in females. This is consistent with a study on male Swiss mice that had high levels of thiobarbituric acid-reactive substances (another measure of lipid peroxidation) in adipose tissue after consuming a high-fat diet [55]. Higher levels of lipid peroxides (Figure 6(b)) in adipose tissue of MarAD males suggest that margarine induces oxidative stress [56]. White adipose tissue is considered the main source of proinflammatory mediators [55]. The upward trend in proinflammatory adipose IL-1β levels in males on the MarAD diet in our study (Figure 6(a)) can imply the proinflammatory effect of margarine. There was a clear sex difference in LOOH levels when mice consumed the experimental diets (Figure 6(b)). In females, when margarine was added to the diet, the level of LOOH in adipose tissue was lower than that in males. According to Ávila et al. [57], during estropause in female mice, ROS levels in the adipose tissue decreased and increased in the liver. In line with this, MarAD-fed females showed significantly higher LOOH levels (Figure 8(a)) in the liver than in controls despite activation of some components of antioxidant defense (Figures 8(d), 8(e)). Continuous consumption of margarine may mimic the effects of estropause in female mice [47], which could explain the difference in LOOH between the sexes.

Both males and females fed margarine had elevated activity of glycolytic enzymes (Figures 7(c), 7(d)). This is consistent with previous studies showing that, compared to mice fed a normal diet, mRNA levels of key glycolytic enzymes (hexokinase, PFK, and PK) were increased in the liver of mice fed HFD [58] and these changes, along with a drop in glycogen levels, are considered to be connected with fatty liver development [58, 59].

### 4.2. Males Are More Sensitive to a Margarine-Restricted Diet

An IF regimen model is used here, as every-other-day fasting is a promising approach to improving health in both humans and laboratory animals [18, 20, 21, 24, 34]. In our study, the MarIF regimen caused significant BM gain in males compared to the standard diet and the MarAD diet. By this, MarIF males consumed fewer food and calories in total but more food/calories in feeding days than other male groups. Calculation of average food intake was made for each day of the experiment, including a day of fasting; that is, food consumed on a day of feeding was divided between 2 days—a feeding day and a fasting day. On a feeding day, males from the MarIF group consumed 1.4-fold more food than males fed MarAD. Obviously, under conditions of prolonged IF, mice tended to compensate for an energy deficit, and the energy consumed was spent sparingly and more efficiently stored in the body of males. Previous studies have also shown that IF led to lower total food intake during the experimental period, even though the food consumption in feeding days could be higher than in *ad libitum* days [22–24, 34]. At the same time, a study by Satta et al. [17], showed that when rats consumed margarine continuously every day, the amount of food consumed did not differ from control values, but when margarine was consumed three times a week, food consumption was higher than in the control group.

Simultaneously with the significant effect on body gain in males, the MarIF regimen seems to induce more changes in metabolism than the MarAD diet. The results may suggest the development of some inflammatory processes in males on the MarIF regimen. Along with the high activity of glycolytic enzymes, we observed a significant decrease in the glycogen content in the liver of the MarIF male. Glucose that is released from the breakdown of glycogen can not only be metabolized by glycolysis in the liver, but also enter the bloodstream and thus may contribute to an increase in blood glucose levels. In addition, we found an increase in the content of storage fats in the liver of male mice, which indicates dysregulation of energy metabolic pathways in the liver of males. These changes could be connected with the increased food intake on the feeding days and a lower ability of males to tolerate high amounts of margarine. Animals use glycogen stores for energy needs and have a compensatory response to fasting in the form of greater lipid accumulation on fasting days.

MarIF females showed largely opposite changes compared to the respective males. In particular, they tended to gain BM more slowly and consume fewer calories daily than their MarAD-fed counterparts. Taking into account that the calculation of food intake was averaged per day of the experiment, including a day of fasting, females in the MarIF group consumed a 2-fold higher amount of food than did the MarAD group. It seems that in MarIF females, higher food intake on feeding days did not provoke significant adverse changes in metabolism. This effect may be due to a modulation of the hormonal status of females. Moreover, MarIF females showed a decrease in activity of proinflammatory MPO and relative levels of neutrophils, lower levels of adipose tissue IL-1β, as well as a downward tendency in IL-1β and total cholesterol in blood and LOOH levels in adipose tissue. The decrease in MPO could be connected with decreasing levels of neutrophils. In addition, lower LOOH and TAG levels, along with activation of some components of antioxidant defense (SOD, GST), were observed in the liver of females on the MarIF regimen as compared to females fed on the MarAD regimen. Together, the results suggest a beneficial effect of IF with margarine on the health status of female mice.

## 5. Conclusions

This study compared the effects of *ad libitum* and IF with the presence/absence of margarine supplements on BM gain and evaluated such parameters as activities of antioxidant and related enzymes, indices of oxidative stress, inflammation, and activities of key glycolytic enzymes and metabolic stores (glycogen, TAGs) in young male and female mice. When mice are given the choice between standard chow and margarine, females prefer eating margarine more than males, but this did not appear to be the reason for BM gain in females. Both sexes showed minimal metabolic disruptions on the MarAD diet, though females exhibited higher liver lipid peroxidation and reduced blood PON activity. Under MarIF, males gained BM faster, with elevated blood glucose, leukocytes, and TAG, but lower liver glycogen and GST activity compared to MarAD-fed males. In contrast, MarIF females showed slower BM gain, improved antioxidant defense, and reduced TAG accumulation compared to MarAD and control groups. Thus, margarine combined with IF benefited female metabolism but adversely affected males. These findings underscore the importance of sex-specific responses in dietary studies, which requires further investigation into the mechanisms behind these differences.

## Data Availability

The data that support the findings of this study are available from the corresponding author upon reasonable request.

## Supporting Information

Supporting Table 1 shows the composition of the laboratory chow that the animals were fed during the experiment.

Supporting File 1 shows the composition of the margarine used for the experimental diets.

Supporting Table 2 shows the estimation of two-way analysis of variance for the measured parameters.

Supporting Figures 1 and 2 show body mass index and the Lee obesity index, respectively, in the experimental groups of mice. Only the MarIF group of females showed significantly higher values of these indices compared with the control groups.

Supporting Figure 3 shows that the MarAD and MarIF groups of mice of both sexes consumed less laboratory chow than the corresponding controls, and the MarIF group consumed less margarine than the MarAD group for the 16 weeks.

Supporting Figure 4 shows that the MarIF groups of both sexes consumed fewer calories from fat than the MarAD group over the 16 weeks.
