# Supplementary material for: Intermittent Fasting Partially Alleviates Dietary Margarine-Induced Morphometrical, Hematological, and Biochemical Changes in Female Mice, but Not in Males
Source: Biochem Res Int. 2025 Jun 18;2025:2163104. doi: 10.1155/bri/2163104 (PMC12197487; doi:10.1155/bri/2163104)
Supplement: Supporting Information — Additional supporting information can be found online in the Supporting Information section. [file 2163104.f1.docx]

**Intermittent fasting partially alleviates dietary margarine-induced morphometrical, hematological, and biochemical changes in mouse females, but not in males**

Viktoriia V. Hurza^1,*^, Maria M. Bayliak^1,*^, Myroslava V. Vatashchuk^1^,

Oksana M. Sorochynska^1^, Maria P. Lylyk^1^, Oleksandra B. Abrat^1^, Dmytro V. Gospodaryov^1^, Kenneth B. Storey^2^, Volodymyr I. Lushchak^1,2,*^

[^1^Department of Biochemistry and Biotechnology, Vasyl Stefanyk Precarpathian National University, Ivano-Frankivsk, Ukraine]

[^2^Research and Development University, Ivano-Frankivsk, Ukraine]

**Conflict of interest statement for production**

The authors declare no conflicts of interest.

**Funding statement for production**

This work was supported by the Ministry of Education and Science of Ukraine [grant numbers #0118U003477 to VIL, #0123U101790 to MMB].

**Supplementary Table 1**

Laboratory chow composition ("Rezon-1" PE, Kyiv, Ukraine): corn, barley, wheat, wheat bran, sunflower oilcake, soybean oilcake, fish flour, feed yeast, milk powder, sunflower oil, salt, vitamins, and Mineral complex.

| Total energy, kcal | 3295 |
| --- | --- |
| Crude protein, % | 22.8-23.0 |
| Crude fiber, % | 5.5-6.5 |
| Raw fat, % | 6.3 |
| Methionine + cystine, % | 0.83 |
| Lysine, % | 1.2 |
| Threonine, % | 0.58 |
| Ca, % | 0.85 |
| P, % | 0.75 |
| Na, % | 0.14 |
| Vitamin A, IU/kg | 15000 |
| Vitamin D3, IU/kg | 2500 |
| Vitamin E, mg/kg | 40 |
| Vitamin K3, mg/kg | 2 |
| Vitamin B1, mg/kg | 4 |
| Vitamin B2, mg/kg | 6 |
| Vitamin B3, mg/kg | 18 |
| Vitamin B4, mg/kg | 400 |
| Vitamin B6, mg/kg | 4 |
| Vitamin B7, mg/kg | 200 |
| Vitamin B9, mg/kg | 2 |
| Vitamin B12, mg/kg | 40 |
| Fe, mg/kg | 100 |
| Zn, mg/kg | 120 |
| I, µg/kg | 280 |
| Co, µg/kg | 1000 |
| Se, µg/kg | 280 |
| Mn, mg/kg | 80 |

**Supplementary File 1**

**Margarine composition** (Soniachnyi Shchedryk, Olkom TM, Kyiv, Ukraine): partially hydrogenated refined deodorized vegetable fats, refined deodorized sunflower oil, drinking water, monoglycerides, soy lecithin, sugar, skim milk powder (0.2%), table salt, potassium sorbate, citric acid, flavoring "Butter-Cream", beta-carotene dye.

**Nutritional value per 100 g:**

Fats – 70.0 g (of which saturated – 12.9-18.2 g)

Carbohydrates – 0.1 g (including sugar – 0.1 g)

Proteins – 0.1 g

Salt – 0.2 g

**Energy value (calorie)** **per 100 g** – 2648 kJ (633 kcal)

**Supplementary Table 2**

Estimates of two-way analysis of variance for measured parameters.

| **Figure** | **Parameter** | ***F*-test** | | ***p*-value** | |
| --- | --- | --- | --- | --- | --- |
|  |  | Diet | Sex | Diet | Sex |
| **Fig. 2C** | Body mass gain | 8.82 | 13.63 | 0.0010 | 0.0009 |
| **Fig. 3I** | Average daily water consumption | 4.07 | 4.52 | 0.0242 | 0.0456 |
| **Fig. 4A** | Total leukocytes | 1.13 | 1.15 | 0.3431 | 0.2971 |
| **Fig. 4B** | Rods neutrophils | 2.31 | 0.70 | 0.1209 | 0.4116 |
| **Fig. 4C** | Segmented neutrophils | 1.99 | 1.66 | 0.1605 | 0.2112 |
| **Fig. 5A** | Free glucose | 3.36 | 0.33 | 0.0499 | 0.5728 |
| **Fig. 5B** | TAG | 4.42 | 3.08 | 0.0223 | 0.0909 |
| **Fig. 5D** | Paraoxonase | 7.19 | 11.10 | 0.0030 | 0.0024 |
| **Fig. 5E** | Myeloperoxidase | 1.37 | 1.00 | 0.2715 | 0.3257 |
| **Fig. 5F** | IL-1β | 1.81 | 8.16 | 0.1845 | 0.0085 |
| **Fig. 6A** | IL-1β | 3.14 | 14.00 | 0.0578 | 0.0008 |
| **Fig. 6B** | LOOH | 0.99 | 28.06 | 0.3820 | <0.0001 |
| **Fig. 7A** | Glycogen | 1.25 | 0.28 | 0.305 | 0.599 |
| **Fig. 7B** | TAG | 16.79 | 7.09 | <0.0001 | 0.0125 |
| **Fig. 7C** | PFK | 4.17 | 123.8 | 0.0252 | <0.0001 |
| **Fig. 7D** | PK | 5.47 | 90.94 | 0.0094 | <0.0001 |
| **Fig. 8A** | LOOH | 15.34 | 31.80 | <0.0001 | <0.0001 |
| **Fig. 8B** | SOD | 2.21 | 18.76 | 0.1311 | 0.0002 |
| **Fig. 8C** | Catalase | 1.42 | 0.23 | 0.2586 | 0.6345 |
| **Fig. 8D** | GST | 0.94 | 41.69 | 0.4032 | <0.0001 |
| **Fig. 8E** | GPx | 3.90 | 39.63 | 0.0313 | <0.0001 |
| **Fig. 8F** | G6PDH | 2.00 | 45.87 | 0.1550 | <0.0001 |

**Supplementary Figure 1**

Body mass index of mice fed standard food *ad libitum* (control), margarine-supplemented food *ad libitum* (MarAD)*,* or margarine-supplemented food with an every-other-day fasting regimen (MarIF) for 16 weeks. *Significantly different from the corresponding control mice with P≤0.05.

**Supplementary Figure 2**

Lee obesity index of mice fed standard food *ad libitum* (control), margarine-supplemented food *ad libitum* (MarAD)*,* or margarine-supplemented food with an every-other-day fasting regimen (MarIF) for 16 weeks. *Significantly different from the corresponding control mice with P≤0.05.

Rodents, including mice, are typically considered obese when their body mass index exceeds 0.35 and Lee index exceeds 0.31.

**Supplementary Figure 3**

Consumption of basic food (A, B) and margarine (C, D) by mice fed standard food *ad libitum* (control), margarine-supplemented food *ad libitum* (MarAD)*,* or margarine-supplemented food with an every-other-day fasting regimen (MarIF) for 16 weeks.

**Supplementary Figure 4**

The number of calories received from fat by mice fed standard food *ad libitum* (control), margarine-supplemented food *ad libitum* (MarAD)*,* or margarine-supplemented food with an every-other-day fasting regimen (MarIF) for 16 weeks. A – by males, B – by females.
